# The Gut Microbiota Affects Corticosterone Production in the Murine Small Intestine

**DOI:** 10.3390/ijms22084229

**Published:** 2021-04-19

**Authors:** Peter Ergang, Karla Vagnerová, Petra Hermanová, Martin Vodička, Michal Jágr, Dagmar Šrůtková, Václav Dvořáček, Tomáš Hudcovic, Jiří Pácha

**Affiliations:** 1 Institute of Physiology, Czech Academy of Sciences, CZ-142 20 Prague, Czech Republic; ergangpeter@gmail.com (P.E.); karla.vagnerova@fgu.cas.cz (K.V.); martin.vodicka@fgu.cas.cz (M.V.); 2 Institute of Microbiology, Czech Academy of Sciences, CZ-549 22 Nový Hrádek, Czech Republic; hermanova@biomed.cas.cz (P.H.); srutkova@biomed.cas.cz (D.Š.); hudcovic@biomed.cas.cz (T.H.); 3 Crop Research Institute, CZ-161 06 Prague, Czech Republic; jagr@vurv.cz (M.J.); dvoracek@vurv.cz (V.D.); 4 Department of Physiology, Faculty of Science, Charles University, CZ-128 00 Prague, Czech Republic

**Keywords:** glucocorticoids, extra-adrenal steroidogenesis, 11β-hydroxysteroid dehydrogenase, intestine, microbiome, anti-CD3 antibody

## Abstract

Glucocorticoids (GCs) are hormones that are released in response to stressors and exhibit many activities, including immunomodulatory and anti-inflammatory activities. They are primarily synthesized in the adrenal gland but are also produced in peripheral tissues via regeneration of adrenal 11-oxo metabolites or by *de novo* synthesis from cholesterol. The present study investigated the influence of the microbiota on *de novo* steroidogenesis and regeneration of corticosterone in the intestine of germ-free (GF) and specific pathogen-free mice challenged with a physical stressor (anti-CD3 antibody i.p. injection). In the small intestine, acute immune stress resulted in increased mRNA levels of the proinflammatory cytokines *IL1β*, *IL6* and *Tnfα* and genes involved in *de novo* steroidogenesis (*Stard3* and *Cyp11a1*), as well as in regeneration of active GCs from their 11-oxo metabolites (*Hsd11b1*). GF mice showed a generally reduced transcriptional response to immune stress, which was accompanied by decreased intestinal corticosterone production and reduced expression of the GC-sensitive marker *Fkbp5*. In contrast, the interaction between stress and the microbiota was not detected at the level of plasma corticosterone or the transcriptional response of adrenal steroidogenic enzymes. The results indicate a differential immune stress-induced intestinal response to proinflammatory stimuli and local corticosterone production driven by the gut microbiota.

## 1. Introduction

Host physiology is significantly influenced by gut microbiota. The microorganisms possess machinery for absorption and metabolism of dietary compounds and secretion of numerous metabolites, which have emerged as important components in the linkage between the microbiota and the host neuro-immuno-endocrine regulatory network [1,2]. However, the mechanisms underpinning this communication remain largely unresolved. The gut microbiota has been implicated in a variety of host reactions, including the response of the hypothalamic–pituitary–adrenal (HPA) axis to stressful stimuli [1]. Studies performed on germ-free (GF) and conventional mice showed that microbiota alters the stress response. GF mice exposed to acute restraint stress exhibited an exaggerated response of the HPA axis with elevated plasma level of corticosterone, and this discrepancy was normalized after colonization of GF mice with commensal bacteria [3]. Similarly, treatment with prebiotics [4] and probiotics [5] attenuated the HPA response to stress.

Stress, and the subsequent activation of the HPA axis, lead to release of glucocorticoid hormones cortisol and corticosterone, which regulate numerous processes such as development, metabolism, behavior and immune functions [6,7]. They are synthesized in the cortex of adrenal glands, and their secretion is under the control of the hypothalamic–pituitary–adrenal axis. However, increasing evidence has indicated the existence of extra-adrenal steroidogenesis, which operates in two different modes, the glucocorticoid de novo synthesis from cholesterol and the regeneration of cortisol and corticosterone from their inert 11-oxo metabolites, which is catalyzed by the enzyme 11β-hydroxysteroid dehydrogenase type 1 [8] Both these pathways were demonstrated in skin [9,10], intestine [11,12,13], lymphoid organs and immune cells [14,15,16,17], although only the first steps of the steroid synthesis pathway were found here.

Numerous studies have shown that immune stress increases the capacity of extra-adrenal organs to produce glucocorticoids. Specifically, activation of either the adaptive immune system by i.p. injection of anti-CD3 antibody or innate immunity by lipopolysaccharides increases local *de novo* synthesis of corticosterone in the intestine and ameliorates intestinal inflammation [11,12,18]. Similarly, acute intestinal inflammation promotes the synthesis of intestinal glucocorticoids [12] and their regeneration via 11HSD1 [17,19]. Nevertheless, the role of microbiota in local synthesis of glucocorticoid hormones in the intestine remains largely unknown, even though some data indicate that: (i) these hormones might participate in the regulation of intestinal immune homeostasis [11,20,21,22,23], (ii) the microbiota is an important factor in modulation of extra-adrenal glucocorticoid steroidogenesis by psychosocial stress [24,25], and (iii) the microbiota could contribute to the regulation of intestinal glucocorticoid generation [21,26,27]. Although understanding whether and how commensal microorganisms modulate the local metabolism of glucocorticoids is important for explaining the physiological role of extra-adrenal glucocorticoids, no studies have investigated the effect of the microbiota on the intestinal metabolism of glucocorticoids in detail. Given that immune stress upregulates intestinal synthesis and regeneration of glucocorticoids, this study investigated whether the gut microbiota is able to control these processes.

## 2. Results

### 2.1. Expression of Glucocorticoid-Related Genes in the Intestine of Anti-CD3 Antibody-Treated Mice

**Figure 1:**
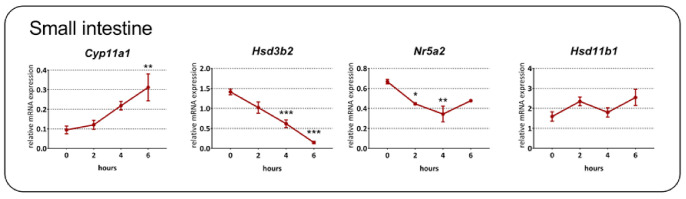
Kinetics of glucocorticoid-related gene expression in the small intestine of specific pathogen-free mice following anti-CD3 antibody injection. *Cyp11a1*, cholesterol side-chain cleavage enzyme; *Hsd3b2*, 3β-hydroxysteroid dehydrogenase type 2; *Nrp5a2*, liver receptor homolog-1, *Hsd11b1*, 11β-hydroxysteroid dehydrogenase type 1. The data are expressed as means ± SEM. Statistical significance at levels * *p* < 0.05, ** *p* < 0.01, *** *p* < 0.001 vs. control group at time zero.

To address the effect of acute immune stress on the expression of genes encoding steroidogenic enzymes, we first studied their temporal regulation in the intestine after *in vivo* T cell activation by anti-CD3 antibody injection. As shown in Figure 1, the expression of *Cyp11a1,* encoding P450scc, the rate-limiting steroidogenic enzyme, was upregulated (one-way ANOVA; F_3,15_ = 6.12, *p* = 0.006) with a significantly increased level 6 h after injection. In contrast, the expression of *Hsd3b2,* encoding the conversion of pregnenolone to progesterone, and *Nr5a2,* encoding a regulatory factor of intestinal extra-adrenal steroidogenesis [28], was downregulated (*Hsd3b2*: F_3,15_ = 30.37, *p* < 0.001; *Nr5a2*: F_3,13_ = 7.98, *p* = 0.003). Surprisingly, we detected *Cyp11b1*, which is responsible for the conversion of 11-deoxycorticosterone to corticosterone, only in several samples at both 4 and 6 h after the injection (*n* = 5 at each time point); in other samples, the levels of this transcript were below the detection limit or were very low (C_p_ > 36). This finding is in agreement with very low expression of *Cyp11b1* in native tissues. By comparison, the expression level of *Hsd11b1,* encoding an enzyme catalyzing the regeneration of corticosterone from 11-dehydrocorticosterone, was relatively high and showed only a tendency to be upregulated by anti-CD3 antibody injection (F_3,16_ = 2.54, *p* = 0.093).

### 2.2. Effect of the Microbiota and Acute Immune Stress on the Expression of Genes Associated with Steroidogenesis in the Small Intestine and Peyer’s Patches

**Figure 2:**
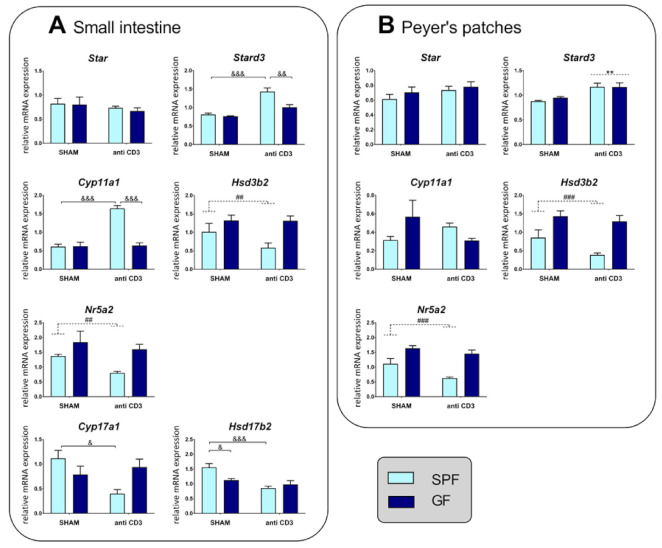
Effect of microbiota and acute immune stress on the expression of steroidogenic enzymes in the small intestine (**A**) and Peyer’s patches (**B**). The abundance of the mRNAs was determined in mice that were treated with the anti-CD3 antibody or saline 4 h beforehand. SPF, specific pathogen-free mice; GF, germ-free mice; SHAM, mice injected with saline; anti-CD3, mice injected with anti-CD3 antibody; *Star,* steroidogenic acute regulatory protein; *Stard3*, functional homolog of *Star*; *Cyp11a1*, cholesterol side-chain cleavage enzyme; *Hsd3b2*, 3β-hydroxysteroid dehydrogenase type 2; *Cyp17a1*, 17,20-desmolase; *Hsd17b2*, 17β-hydroxysteroid dehydrogenase type 2; *Nrp5a2*, liver receptor homolog-1. The data are expressed as means ± SEM. Where an interaction effect was observed, the ampersand sign indicates a significant difference (^&^ *p* < 0.05, ^&&^ *p* < 0.01, ^&&&^ *p* < 0.001). Where no interaction effect was observed, a main effect of microbiota has been marked by placing a dashed horizontal line with a hash sign (^##^ *p* < 0.01, ^###^ *p* < 0.001) above the bars for the SPF groups, whereas a main effect of stress has been marked by placing a dashed horizontal line with an asterisk (** *p* < 0.01) above the bars for the stress-exposed groups.

To establish the impact of the microbiota on the induction of local extra-adrenal pathways of glucocorticoid generation during acute immune stress, we examined the expression of steroidogenic enzymes and factors participating in *de novo* steroidogenesis in the intestine. Two-way ANOVA revealed that both the microbiota and immune stress modulated the expression of *Stard3* and *Cyp11a1,* and that there was a strong interaction between these two factors (Table S1). As shown in Figure 2A, acute immune stress upregulated *Stard3* and *Cyp11a1* in SPF but not GF mice, whereas the expression of *Star* was not modulated by either the microbiota or immune stress, and the absence of the microbiota led to upregulation of *Hsd3b2.* An interaction between the microbiota and stress was also observed in the regulation of genes encoding enzymes that catalyze the conversion of pregnenolone and progesterone to androgens (Table S1) and whose synthetic pathway was described in the gastrointestinal tract [29]. Namely, the expression of *Cyp17a1* and *Hsd17b2* was downregulated by immune stress in SPF but not GF mice (Figure 2A). In contrast, *Nr5a2,* encoding a regulator of extra-adrenal glucocorticoid synthesis in the intestine [28] was not significantly affected by stress, although it was decreased in the presence of the microbiota, similar to *Hsd3b2*.

We next examined the impact of the microbiota and immune stress on Peyer’s patches, which are considered to be the inductive sites for mucosal B and T cells and are very sensitive to the presence and absence of the microbiota [30]. In contrast to the small intestine, *Cyp11a1* expression did not depend on the microbiota and immune stress, and *Stard3* upregulation by stress was independent of the microbiota (Figure 2B). The effects of stress and the microbiota on the expression of *Star*, *Hsd3b2* and *Nr5a2* were similar to those in the small intestine (Figure 2B, Table S1).

### 2.3. Expression Levels of 11β-Hydroxysteroid Dehydrogenase Type 1 and Type 2 in the Small Intestine and Peyer’s Patches 

**Figure 3:**
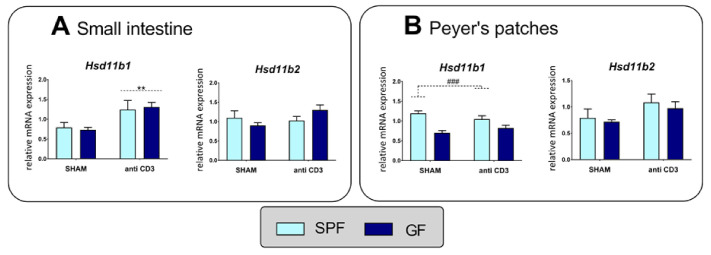
Effect of microbiota and acute immune stress on the expression of 11β-hydroxysteroid dehydrogenase type 1 (*Hsd11b1*) and 11β-hydroxysteroid dehydrogenase type 2 (*Hsd11b2*) in the small intestine (**A**) and Peyer’s patches (**B**). The data are expressed as means ± SEM. Main effect of microbiota has been marked by placing a dashed horizontal line with a hash sign (^###^ *p* < 0.001) above the bars for the SPF groups, whereas a main effect of stress has been marked by placing a dashed horizontal line with an asterisk (** *p* < 0.01) above the bars for the stress-exposed groups.

To determine the effect of immune stress and the microbiota on the regeneration of corticosteroids, we quantified *Hsd11b1* and *Hsd11b2* expression. In both the intestine and Peyer’s patches, neither stress nor the microbiota affected the expression of *Hsd11b2,* which encodes a conversion of corticosterone to inactive 11-dehydrocorticosterone. By contrast, the expression level of *Hsd11b1,* which catalyzes the reduction of 11-dehydrocorticosterone to corticosterone, was stimulated by immune stress in the small intestine but not in Peyer’s patches, where the transcript level depended on the microbiota (Figure 3, Table S1).

### 2.4. Production of Steroids in the Intestine 

**Figure 4:**
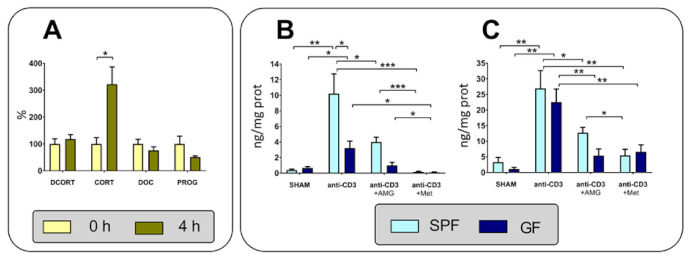
*Ex vivo* steroid production by intestinal tissue. (**A**) Changes in the levels of 11-dehydrocorticosterone (DCORT), corticosterone (CORT), progesterone (PROG) and 11-deoxycorticosterone (DOC) in tissue explants after 4 h incubation in tissue culture. (**B**) Effect of the anti-CD3 antibody, aminoglutethimide (AMG) and metyrapone (Met) on the level of corticosterone. (**C**) Effect of the anti-CD3 antibody, AMG and Met on the level of 11-dehydrocorticosterone. Specific pathogen-free (SPF) and germ-free (GF) mice were injected with saline (SHAM) or the anti-CD3 antibody and sacrificed 4 h later, and the intestinal fragments were cultured *ex vivo* in the presence or absence of AMG or Met for 4 h. * *p* < 0.05; ** *p* < 0.01; *** *p* < 0.001.

To confirm whether the changes in the expression of genes encoding enzymes of steroid synthesis from cholesterol to corticosterone and those participating in regeneration of corticosterone from 11-dehydrocorticosterone are confirmed at the level of local metabolism, we further determined the *ex vivo* steroid production in the intestine of GF and SPF mice exposed to the anti-CD3 challenge. LC-MS/MS analysis identified 11-dehydrocorticosterone and corticosterone and much lower levels of progesterone and 11-deoxycorticosterone in the samples of the small intestine; other steroids, such as dehydroepiandrosterone, androstendiol, androstendione and testosterone, were not found. As shown in Figure 4A, the content of corticosterone in the intestine explants increased more than three times after 4 h, whereas the levels of 11-dehydrocorticosterone, progesterone and deoxycorticosterone were not changed.

To further understand glucocorticoid synthesis, we investigated the effects of inhibition of steroidogenic enzymes by AMG and metyrapone on the production of corticosterone and 11-dehydrocorticosterone. AMG blocks the first step of steroid biogenesis by inhibiting the cholesterol side-chain cleavage enzyme (*CYP11a1*), whereas metyrapone inhibits glucocorticoid synthesis by inhibiting 11β-hydroxylase (*CYP11b1*) [31] and regeneration of corticosterone from 11-dehydrocorticosteroen by inhibiting 11β-hydroxysteroid dehydrogenase type 1 (11HSD1), but it does not inhibit oxidation of corticosterone via 11β-hydroxysteroid dehydrogenase type 2 (11HSD2) [32,33]. Only small levels of corticosterone and 11-dehydrocorticosterone were found in intestinal fragments of unstimulated mice, but these levels were significantly enhanced after stimulation with anti-CD3 antibodies (Figure 4B,C). The production of corticosterone after the anti-CD3 challenge was significantly higher in SPF mice than in GF mice (Figure 4B). Both corticosterone and 11-dehydrocorticosterone production was reduced in the presence of AMG and even more reduced in the presence of metyrapone, and this effect was more obvious in SPF mice than in GF mice. Metyrapone showed a stronger inhibitory effect on corticosterone than 11-dehydrocorticosterone (Figure 4B,C).

### 2.5. Response of Cytokines and HPA Axis to Acute Anti-CD3 Challenge 

**Figure 5:**
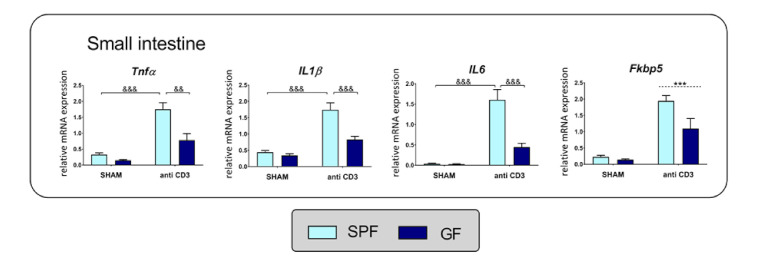
Anti-CD3 antibody treatment and microbiota alter the expression of cytokine and glucocorticoid-responsive genes. Gene expression of tumor necrosis factor α (*Tnfα*), interleukin 1β (*IL1β*), interleukin 6 (*IL6*) and cochaperone FK506 binding protein 5 (*Fkbp5*) was measured by quantitative PCR in the small intestine of specific pathogen-free (SPF) and germ-free (GF) mice 4 h after *in vivo* treatment with the anti-CD3 antibody or saline (SHAM). The data are expressed as means ± SEM. The ampersand sign indicates a significant difference (^&&^ *p* < 0.01, ^&&&^ *p* < 0.001) of microbiota*stress interaction effect. Where no interaction was observed, a main effect of stress has been marked by placing a dashed horizontal line with an asterisk (*** *p* < 0.001) above the bars for the stress-exposed groups.

The proinflammatory cytokine TNFα has been previously identified as a stimulator of intestinal steroidogenesis [12,18] and glucocorticoid regeneration [17,34]; therefore, we examined the effects of the microbiota and immune stress on proinflammatory cytokines in the intestine of SPF and GF mice. Similar to *Cyp11a1* and *Stard3*, ANOVA indicated that the expression of genes encoding proinflammatory cytokines TNFα, IL-1β and IL-6 depended on the microbiota, stress and the interaction of microbiota*stress (Table S1). As shown in Figure 5, acute anti-CD3 challenge upregulated the expression of cytokines, but this effect depended strongly on the presence of the microbiota. A similar expression pattern was observed for *Fkbp5*, a known glucocorticoid target gene and a strong glucocorticoid-sensitive marker [35], which was upregulated by anti-CD3 challenge and showed a tendency to be upregulated by the microbiota (Table S1, Figure 5).

**Figure 6:**
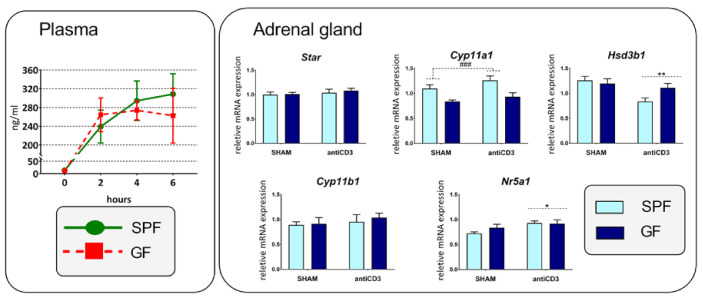
Effect of microbiota and acute immune stress on plasma concentration of corticosterone and the expression of genes participating in adrenal steroidogenesis. SPF, specific pathogen-free mice; GF, germ-free mice; SHAM, mice injected with saline; anti-CD3, mice injected with anti-CD3 antibody; *Star*, steroidogenic acute regulatory protein; *Cyp11a1*, cholesterol side-chain cleavage enzyme; *Hsd3b1*, 3β-hydroxysteroid dehydrogenase type 1; *Cyp11b1*, 11β-hydroxylase; *Nr5a1*, steroidogenic factor 1. The data are expressed as means ± SEM. Two-way ANOVA revealed main effect of time on plasma concentration of corticosterone (microbiota: NS; time: F_3,32_ = 31,98, *p* < 0.001; microbiota*time: NS), microbiota on *Cyp11a1* (microbiota: F_1,34_ = 17.14, *p* < 0.001; stress: NS, microbiota*stress: NS), and anti-CD3 stress on *Nr5a1* (microbiota: NS; stress: F_1,34_ = 5.85, *p* = 0.021; microbiota*stress: NS) and *Hsd3b1* (microbiota: NS; stress: F_1,34_ = 8.91, *p* = 0.005; microbiota*stress: NS). A main effect of microbiota has been marked by placing a dashed horizontal line with a hash sign (^###^ *p* < 0.001) above the bars for the SPF groups, whereas a main effect of stress has been marked by placing a dashed horizontal line with an asterisk (* *p* < 0.05, ** *p* < 0.01) above the bars for the stress-exposed groups.

We next examined whether anti-CD3-mediated activation of the HPA axis is microbiome-dependent by monitoring the plasma concentration of corticosterone and the expression of selective adrenal genes related to the stress response and steroidogenesis. The anti-CD3 antibody extensively upregulated the plasma level of corticosterone (Figure 6) without any significant differences between SPF and GF mice. Similarly, the expression of adrenal gland steroidogenesis genes did not show any interaction between immune stress and the microbiota. As shown in Figure 6, *Cyp11a1* expression was significantly increased in the presence of the microbiota but not by immune stress, an effect found in the case of intestinal *Cyp11a1*. In contrast, immune stress weakly elevated the expression of *Nr5a1*, a critical transcription factor of a variety of adrenal steroidogenic enzymes [36], and downregulated *Hsd3b1*. The expression of *Star* and *Cyp11b1* did not depend on either the microbiota or anti-CD3 challenge.

### 2.6. Immune Response to Anti-CD3 Antibody Injection

**Figure 7:**
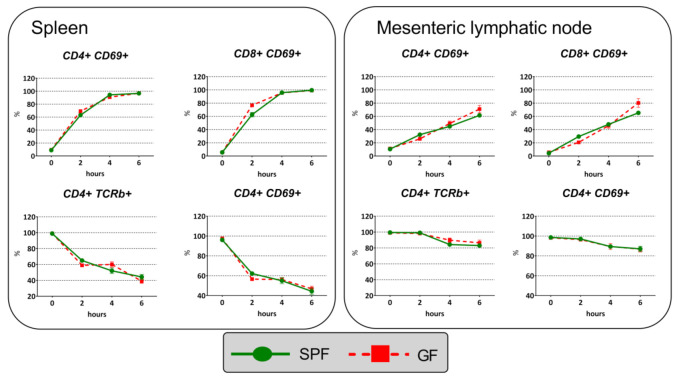
Changes in the expression of the T-cell activation markers CD69 and TCRβ in CD4^+^ and CD8^+^ subpopulations of CD3^+^ T cells isolated from spleen and mesenteric lymphatic nodes after their activation via anti-CD3 crosslinking of the T-cell receptor. The data are shown as mean percentages ± SEM. Two-way ANOVA identified the effect of time (*p* < 10^−6^), but not the effect of microbiota (*p* > 0.05) or interaction time*microbiota (*p* > 0.05).

To determine whether *in vivo* T-cell activation via anti-CD3 crosslinking of the T-cell receptor results in similar activation and proportion changes in SPF and GF mice, we measured activation of CD4^+^ and CD8^+^ T cells in splenocytes and MLN cells using activation markers CD69 and TCRβ. The results showed that the percentages of the CD4^+^CD69^+^ T cell and CD8^+^CD69^+^ T cell subpopulations of splenocytes and MLN cells progressively increased in a time-dependent manner (Figure 7). In contrast, the frequencies of CD4^+^TCRβ^+^ T cells and CD8^+^TCRβ^+^ T cells gradually decreased; however, this effect was more obvious in splenic than MLN populations. Using two-way ANOVA with the factors time and microbiota, we found that the time-dependent changes in the frequencies of the CD4^+^CD69^+^/CD8^+^CD69^+^ and CD4^+^TCRβ^+^/CD8^+^TCRβ^+^ subpopulations induced by anti-CD3 administration depended strongly on time (*p* < 10^−6^) but did not depend on the presence of the microbiota, and that the frequencies of cell populations progressively increased or reduced were not different across the time points between GF and SPF mice.

## 3. Discussion

In this study, we addressed the question of whether the microbiota is capable of modulating the local production of glucocorticoids. The microbiota impacts the systemic glucocorticoid response to stress [3,37,38] or injection to chemotherapeutic stress [39]. However, whether the microbiota is involved in local corticosterone synthesis and regeneration is not clear, although under basal conditions, the microbiota may contribute to the regulation of intestinal glucocorticoid synthesis [27], and activation of immune cells by anti-CD3 antibody leads to upregulation of intestinal *de novo* synthesis [11] and regeneration of corticosterone [16]. In this report, we showed that the intestine of mice represents an extra-adrenal site of corticosterone production, which is activated by immune stress and is more active in SPF mice than in GF mice. First, we demonstrated the presence of detectable amounts of mRNA transcripts of most genes encoding the enzymes and regulatory proteins of corticosteroid synthesis. Second, we confirmed *ex vivo* intestinal production of corticosterone and 11-dehydrocorticosterone, which was elevated in response to immune stress. Third, we identified nonhomogeneous response induced by immune stress and/or the microbiota in GF and SPF mice.

We found constitutive expression of genes encoding enzymes mediating corticosterone regeneration (*Hsd11b1*) and inactivation (*Hsd11b2*), and *de novo* corticosterone synthesis both in SPF and GF mice, although the expression of *Cyp11b1* was very low and often undetectable (found at low levels only in some samples, C_p_ > 36) not only in untreated but also in immune-activated mice, whose intestines produced AMG-blockable corticosterone. The reason that *Cyp11b1* was often undetectable in our mice but was detected in some other studies [11,12] is unknown, although we cannot exclude strain differences (BALB/c vs. C57BL/6) or the environmental factors/composition of the microbiome in the specific animal facilities. Mukherji et al. [27] demonstrated downregulation of intestinal *Cyp11a1* in conventional mice in comparison with GF mice and mice with antibiotic-induced microbiota depletion, whereas in the study of Ballegeer et al. [21], *Cyp11a1* expression was reduced in mice subjected to antibiotics. Both the microbiota and anti-CD3 antibody treatment impacted the expression profile of genes encoding steroidogenic enzymes, and in the cases of *Cyp11a1*, *Cyp17a1* and *Hsd17b2,* the response to immune stress was microbiota-dependent. In SPF mice, immune stress downregulated the expression of genes of the C_19_ branch but upregulated the expression of *Cyp11a1,* and did not have the same effect on *Hsd3b2* and the transcription factor *Nr5a2*. This factor is considered an important factor in intestinal corticosterone synthesis [28], including in transcriptional regulation of 3β-hydroxysteroid dehydrogenases [40]. These findings indicate that the initial trigger for upregulation of *Cyp11a1* during immune stress is not *Nr5a2,* but probably proinflammatory cytokines, whose expression profiles were similar to those of *Cyp11a1* and *Stard3*. This hypothesis is in agreement with the in vitro induction of *Cyp11a1* by proinflammatory cytokines reported recently [15]. The absence of expected changes in the expression of *Nr5a2,* encoding the transcription factor regulating intestinal *Cyp11a1* and *Cyp11b1* [41], supports the existence of an *Nr5a2*-independent signaling pathway regulating intestinal steroidogenesis [18].

In contrast to the effect of the microbiota and immune stress on the expression of steroidogenesis genes, the expression of *Hsd11b2,* encoding the corticosterone-inactivating enzyme 11HSD2, was not changed by the microbiota or immune stress, and *Hsd11b1,* encoding the corticosterone-activating enzyme 11HSD1, was upregulated by immune stress regardless of the presence or absence of the microbiota. Upregulation of *Hsd11b1* and increased conversion of 11-dehydrocorticosterone to corticosterone by 11HSD1 is typical for intestinal inflammation [42,43]. Considering the localization of 11HSD1 exclusively to nonepithelial cells, including intraepithelial leukocytes and lamina propria cells [13,17], the elevated *Hsd11b1* transcript in the murine intestine of anti-CD3 antibody-treated mice reflects the response of gut immune cells, which differs from extra-adrenal steroidogenesis localized to the intestinal epithelium [11,41]. The mechanism that underlies the effect of stress on the regeneration of corticosterone is currently unknown. Cytokines are important modulators of *Hsd11b1* and the 11oxo-reductase activity of 11HSD1 [17,34,44]; however, in our experiments, anti-CD3 antibody treatment and the microbiota showed a strong interaction in the upregulation of the expression of proinflammatory cytokines, which was not observed in the case of *Hsd11b1*. Similarly, the expression of *Hsd11b1* in Peyer’s patches was not changed after anti-CD3 antibody treatment, although the expression of proinflammatory cytokines was upregulated (data not shown), and lymphocyte activation by anti-CD3 challenge upregulated the expression of *Hsd11b1* and 11HSD1 [16]. This finding is particularly interesting because it indicates that there may be differences in the control of local regeneration of corticosterone in the immune cells of gut-associated lymphoid tissue, such as Peyer’s patches, and in the effector sites of the intestinal immune system in the lamina propria and intestinal epithelium/intraepithelial lymphocytes.

To provide direct evidence that corticosterone is synthesized in the intestine of SPF and GF mice, the tissue was incubated ex vivo and steroid content was analyzed using LC-MS/MS. The observation that the concentration of corticosterone was increased greater than three times after 4 h of incubation of the intestine *ex vivo* demonstrated that the tissue is capable of synthesizing considerable amounts of corticosterone, and that anti-CD3 challenge increases the intestinal production of corticosterone. These results are in accordance with previous studies showing that the intestine secretes corticosterone after immune stimulation [11,20]. In addition, immune stimulation upregulated *Cyp11a1,* encoding the key enzyme of steroidogenesis, and *Hsd11b1,* responsible for corticosterone regeneration from 11-dehydrocorticosterone. These findings suggest that two pathways may lead to increased production of corticosterone, *de novo* synthesis and regeneration of corticosterone. Decreased production of corticosterone and 11-dehydrocorticosterone by AMG confirms the presence of CYP11a1 activity and similarly the inhibitory effect of metyrapone the presence of 11β-hydroxylase (CYP11B1) and/or 11HSD1 enzymatic activity [31,32,33]. However, the inhibitory effect of metyrapone on corticosterone concentration was more distinct than that on 11-dehydrocorticosterone. Therefore, we hypothesize that tissue explants generate corticosterone from cholesterol by the steroidogenic pathway and from 11-dehydrocorticosterone via reduction catalyzed by 11HSD1, while corticosterone is oxidized via 11HSD2, an enzyme localized in enterocytes [13]. The presence of metyrapone blocks *de novo* synthesis and regeneration of corticosterone, but not oxidation of corticosterone to 11-dehydrocorticosterone because the blocker inhibits 11HSD1 but not 11HSD2 [32]. Therefore, we assume that conversion of 11-dehydrocorticosterone may contribute considerably to the local production of corticosterone in the intestine.

Furthermore, the present study showed that the generation of corticosterone was higher in the intestine of immune-activated SPF mice than in that of GF mice, although the plasma concentration of corticosterone was the same in both groups. This finding is in agreement with stronger upregulation of intestinal *Cyp11a1* and *Stard3,* encoding a rate-limiting enzyme in steroid synthesis and a mitochondrial cholesterol importer [45], respectively, and *Fkbp5*, a sensitive marker of glucocorticoid exposure [35], in SPF than GF mice. Further studies will have to be conducted to reveal the mechanisms of discrepancy between the production of corticosterone in SPF and GF mice. Similar to our experiments, the recent study of Ballegeer et al. [21] demonstrated a reduction in *ex vivo* corticosterone production by intestinal explants of mice treated with antibiotics. Our data indicate that the difference in local production of corticosterone cannot be explained by the different responsiveness of the immune system of SPF and GF mice to T-cell activation by anti-CD3 challenge. However, this might be related to the increased upregulation of TNFαa transcripts observed in our experiments. Administration of anti-CD3 antibody leads to higher TNFα levels in the jejunum in SPF mice than in GF mice [46]; thus, TNFα-dependent regulation of intestinal steroidogenesis [18] and glucocorticoid regeneration [17,34] might provide indirect evidence for the role of TNFα in the reduced corticosterone levels in GF mice.

Taken together, the data presented here demonstrate that microbial colonization shapes the intestinal extra-adrenal production of glucocorticoids in response to activation of the adaptive immune system. Furthermore, our findings suggest that amplification of corticosterone synthesis by immune stress reflects upregulation of both the *de novo* synthesis and regeneration pathways.

## 4. Materials and Methods

### 4.1. Animals, Treatment and Sample Collection

The experiments were performed on 9- to 13-week-old germ-free (GF) and specific pathogen-free (SPF) male BALB/c mice (Institute of Microbiology, Nový Hrádek, Czech Republic), which were maintained on a 12 h light/dark cycle and were given free access to autoclaved tap water and an irradiated (50 kGy) sterile pellet diet, Altromin 1414 (Altromin, Lage, Germany). Four groups of animals were used: GF mice injected with saline (*n* = 10), GF mice injected with anti-CD3 antibody (*n* = 10), SPF mice injected with saline (*n* = 9) and SPF mice injected with anti-CD3 antibody (*n* = 9). The GF animals were kept under sterile conditions in Trexler-type isolators since birth, and their sterility was assessed every week by microbial cultivation and staining methods. The sterility of the isolator was tested routinely by aerobic and anaerobic cultivation of mouse feces and swabs from the isolators. The GF status of the mice was further confirmed by the cecal size, weight and bacterial DNA content when the GF mice were used in the experiments. To ensure equal conditions for all animals during the experiment, the SPF mice were kept under the same conditions as GF mice, i.e., they were fed a sterile diet, provided autoclaved drinking water, reared on sterile bedding and manipulated by the same staff as the GF mice. Twenty-four hours before the experiments, all mice were transferred into sterile “individually ventilated cages” equipped with a filter system (IVC box; Tecniplast S.p.A., Buguggiate, Italy) to minimize contamination of GF mice with microorganisms and to ensure identical conditions for both GF and SPF mice.

The immune system and intestinal steroidogenesis were activated according to the method of Cima et al. [11]. Shortly, the GF and SPF mice received i.p. injection of 100 µL of saline containing 10 µg of the anti-CD3 antibody (145-2C11, eBioscience, purchased from Thermo Fisher Scientific, Waltham, MA, USA, cat. no. 16-0031-85) in a laminar flow hood and were left in a sterile IVC box. Control animals were treated with saline only. The animals were anesthetized at different time points (for 2, 4 or 6 h) with isoflurane vapor, blood was collected by cardiac puncture and centrifuged, and the plasma was stored at −80 °C. Anesthetized mice were decapitated, and the adrenal gland, small intestine, Peyer’s patches, spleen and mesenteric lymph nodes (MLNs) were harvested. The spleen and MLNs were used for the preparation of single-cell suspensions. The adrenal gland, Peyer’s patches and part of the small intestine were frozen in liquid nitrogen for assessment of mRNA expression, and the remaining intestine was used for preparation of intestinal tissue explants. The intestinal samples were collected from the middle part of the intestine. To minimize the effect of diurnal factors, the mice were injected with the anti-CD3 antibody and saline between 9:00 and 11:00.

### 4.2. Sample Preparation and Real-Time PCR

Total RNA was extracted from the adrenal gland, Peyer’s patches and small intestine using a commercially available kit (RNeasy Plus Universal Mini Kit, Qiagen, Hilden, Germany) according to the manufacturer’s instructions and quantified by spectrophotometry using a NanoDrop ONE spectrophotometer. First-strand cDNA was prepared from total RNA using random hexamers and a High-Capacity cDNA Reverse Transcription Kit (Life Technologies, Carlsbad, CA, USA). Quantitative RT-PCR was carried out using a LightCycler 480 PCR System (Roche Diagnostic GmbH, Mannheim, Germany), 5x Hot FIREpol Probe qPCR Mix Plus (ROX) (Solis BioDyne, Tartu, Estonia) and probes specific for the studied transcripts (TaqMan Assays, Life Technologies). The following assays were used: steroidogenic acute regulatory protein StAR/STARD1 (*Star*, Mm00441558_m1); StAR related lipid transfer domain containing 3 (STARD3), a functional homolog of StAR (*Stard3*, Mm00445524_m1); cholesterol side-chain cleavage enzyme, P450scc (*Cyp11a1*, Mm00490735_m1); 3β-hydroxysteroid dehydrogenase type 1, the major isoform expressed in adrenal gland (*Hsd3b1*, Mm01261921_mH); 3β-hydroxysteroid dehydrogenase type 2, the isoform predominantly expressed in extra-adrenal tissues (*Hsd3b2*, Mm00462685_m1); 11β-hydroxylase (*Cyp11b1*, Mm01204952_m1); 17,20-desmolase (*Cyp17a1*, Mm00484040); 17β-hydroxysteroid dehydrogenase type 2 (*Hsd17b2*, Mm00500430); steroidogenic factor-1 (*Nr5a1*, Mm00446826-m1); liver receptor homolog 1 (*Nr5a2,* Mm00446088); 11β-hydroxysteroid dehydrogenase type 1 (*Hsd11b1*, Mm00476182); 11β-hydroxysteroid dehydrogenase type 2 (*Hsd11b2*, Mm01251104); tumor necrosis factor α (*Tnfα,* Mm00443258_m1), interleukin 1β (*IL1β,* Mm00434228); interleukin 6 (*IL6,* Mm00446190); and cochaperone FK506 binding protein 5 (*Fkbp5*, Mm00487401_m1). The quantity of the PCR product was determined using the standard curve method with serial 3-fold dilutions of the mixed cDNA sample, and the expression levels of the genes of interest were calculated relative to the geometric mean of the reference genes in each sample. The expression levels of the genes of interest in the adrenal gland were normalized to those of *Ppib* (peptidylprolyl isomerase B, cyclophilin B, Mm00478295_m1) and *Sdha* (succinate dehydrogenase subunit A, Mm01352366_m1), those in the small intestine to *Sdha* and *Tbp* (TATA-box binding protein, Mm00446973_m1) and those in Peyer’s patches to *Ppib* and *Hprt1* (hypoxanthine-guanine phosphoribosyltransferase 1, Mm01545399-m1). These reference genes provided the highest stability in the panel of 12 potential reference genes tested.

### 4.3. Steroidogenesis in Intestinal Explants 

Immediately after collection, the intestine free of Peyer’s patches was cut into 3–5 mm fragments and washed in DMEM containing 2% charcoal/dextranT70-stripped fetal bovine serum. The tissue fragments were incubated in carbogen gas atmosphere (95% O_2_ and 5% CO_2_) for 4 h (2, 4 or 6 h in pilot experiments) at 37 °C in 24-well plates in DMEM containing 10% charcoal/dextranT70-stripped fetal bovine serum [11]. For inhibition of steroidogenesis, we used metyrapone (50 µM), a blocker of 11β-hydroxylase and 11β-hydroxysteroid dehydrogenase type 1, and aminoglutethimide (AMG, 50 µM), a blocker of P450scc, which converts cholesterol to pregnenolone [31,32]. The incubation of explants was stopped by cooling on ice, the medium was separated from the tissue fragments and both samples were stored at −80 °C. Before the extraction of steroids, the tissue fragments were homogenized in 1 mL of ice-cold water with Polytron homogenizer, and 10 µL of the homogenate was used for protein quantification using the BCA method. The remaining aliquot of homogenate and the incubation medium were polled and extracted twice with 2 mL of tert-butyl methyl ether. The solution was separated by centrifugation at 1500 rpm for 20 min and frozen. The organic upper phase was transferred to a clean tube, evaporated in a nitrogen stream at 40 °C and stored at −80 °C.

### 4.4. Quantification of Steroids Using MS Analysis

The dried extracts of tissue explants were dissolved in 150 µL of 100% methanol containing steroids (corticosterone-d_4_, cortisone-d_8_ and progesterone-d_9,_ final concentration 100 ng/mL; all from Sigma-Aldrich) were used as internal standards as internal standards in Eppendorf tubes using an ultrasonic bath, centrifuged (15 min; 13,500 rpm; 25 °C) and filtered through 0.2 µm PVDF spin filters (Thermo Fisher Scientific, Rockwood, TN, USA). The extracts were transferred to vials and stored at −18 °C prior to UHPLC-ESI-MS/MS analysis.

Analysis and detection were performed using a Dionex UltiMate 3000 UHPLC system (Dionex Softron GmbH, Germering, Germany) and a quadrupole/orbital ion trap Q Exactive mass spectrometer (Thermo Fisher Scientific, San Jose, CA, USA) equipped with a heated electrospray ionization source (HESI-II) and Xcalibur software, version 4.0. Chromatographic separation was performed with a reversed-phase Kinetex XB-C18 2.1 × 100 mm, 2.6 µm column from Phenomenex (Torrance, CA, USA) using gradient elution with 0.2% formic acid in water (*v*/*v*, A) and 0.2% formic acid in methanol (*v*/*v*, B). Separation was started by running the system with 70% of solvent A + 30% of solvent B, which was held isocratic for 2 min, followed by gradient elution to 5% A + 95% B in 11 min. The column was then eluted for 2 min at 100% of B. Equilibration before the next run was achieved by rinsing the column with 70% A + 30% B for 2 min. The total analysis time was 15 min. The column was maintained at 40 °C at a flow rate of 0.35 mL/min, and the injection volume was 3 µL.

MS detection was performed using electrospray ionization in positive ion mode with the following source conditions: spray voltage 2.5 kV; sheath gas flow 49 AU; auxiliary gas flow 12 AU and sweep gas flow 2 AU; capillary temperature 260 °C; heater temperature 419 °C. Nitrogen was used as the sheath, auxiliary and sweep gas. Parallel reaction monitoring (PRM) was used for the quantification of the steroids. The precursor ions in the inclusion list were isolated within the retention time window ±60 s, filtered by the quadrupole in the isolation window (target m/z ± 0.8 m/z), and fragmented in an HCD collision cell. The accuracy and calibration of the Q Exactive Orbitrap LC-MS/MS was checked using a reference standard mixture obtained from Thermo Fisher Scientific. Data were evaluated by Quan/Qual Browser Xcalibur software, version 4.0. Identification of steroid compounds in samples was based on their retention times relative to those of the authentic standards and on mass spectral data (accurate mass determination generating elemental composition and fragmentation patterns of a molecular ion) obtained by LC-MS/MS, which were compared with those described in previous studies [47,48]. Quantitative analysis was performed on the basis of calibration curves.

### 4.5. Corticosterone Assay

The plasma concentration of corticosterone was measured using a commercially available corticosterone rat/mouse ELISA kit (LDN GmbH, Nordhorn, Germany) according to the manufacturer’s instructions. The samples were measured in a single run to prevent interassay variability.

### 4.6. Flow Cytometric Analysis of Splenocytes and MLN Cells

Single-cell suspensions of spleen and MLNs were obtained by mechanical dissociation through a 70 µm cell strainer, and red blood cells were depleted by ACK lysis buffer. The dissociated cells were washed twice in FACS buffer (PBS + 5% FBS). The cells were counted and labeled with fluorophore-conjugated antimouse antibodies (all purchased from eBioscience), and phenotypic analysis was performed on a FACSCalibur flow cytometer using FlowJo software (Tree Star Inc., Ashland, OR, USA) for data analysis. The following antibodies were used: FITC anti-CD4 (Rm4-5), APC anti-CD8a (53-6.4), PE anti-CD69 (H1.2F3), PerCP-Cyanine5.5 anti-TCR beta (H57-597), FITC rat IgG2a,κ, APC rat IgG2a,κ, PE Armenian hamster IgG and PerCP-Cyanine5.5 Armenian hamster IgG.

### 4.7. Statistical Analysis

The data are presented as the mean ± SEM values. Statistical analyses were carried out with GraphPad Prism 6 software (GraphPad, La Jolla, CA, USA). Shapiro–Wilk’s test was employed to test normal distribution of all acquired data sets, and the normally distributed data sets were subsequently analyzed by either one-way or two-way analysis of variance (ANOVA) followed by *post hoc* Tukey’s test in the instances where ANOVA yielded a significant effect of main factor or interaction. The effects of steroidogenic enzyme inhibitors were assessed using unpaired two-tailed Student’s *t*-test. Values of *p* less than 0.05 were considered statistically significant.

## Supplementary Materials

The following is available online at https://www.mdpi.com/article/10.3390/ijms22084229/s1: Table S1: Results of two-way ANOVA comparing the effects of microbiota and acute immune stress in the small intestine and Peyer’s patches.
