# Supplementary material for: The Gut Microbiota Affects Corticosterone Production in the Murine Small Intestine
Source: Int J Mol Sci. 2021 Apr 19;22(8):4229. doi: 10.3390/ijms22084229 (PMC8073041; doi:10.3390/ijms22084229)
Supplement: Supplementary file 1 [file ijms-22-04229-s001.zip › ijms-1179149-supplementary.pdf]

Table S1. Results of two-way ANOVA comparing the effects of microbiota and acute immune stress in the small intestine and Peyer's patches

	Df	Microbiota	Stress	Interaction
		Small intestine		
Star	1,22	0.685 (0.17)	0.277 (1.25)	0.821 (0.05)
Stard3	1,23	**0.005** (9.57)	< **0.001** (31,86)	**0.023** (5.97)
Cyp11a1	1,23	< **0.001** (35.92)	< **0.001** (40.1)	< **0.001** (37.47)
Hsd3b2	1,24	**0.003** (10.74)	0.178 (1.93)	0.193 (1.48)
Cyp17a1	1,23	0.482 (0.51)	0.069 (3.63)	**0.007** (8.64)
Hsd17b2	1,24	0.142 (2.31)	< **0.001** (17.78)	**0.011** (7.71)
Nr5a2	1,22	**0.005** (9.53)	0.061 (3.92)	0.435 (0.63)
Hsd11b1	1,24	0.991 (< 0.01)	**0.004** (10.36)	0.707 (0.14)
Hsd11b2	1,24	0.760 (0.10)	0.216 (1.61)	0.079 (3.36)
IL1β	1,22	**0.001** (14.53)	< **0.001** (45.03)	**0.006** (9.41)
Tnfα	1,22	**0.002** (12.46)	< **0.001** (39.92)	**0.023** (5.95)
IL6	1,21	**0.005** (10.06)	< **0.001**(29.05)	**0.005** (9.72)
Fkbp5	1,24	**0.032** (5.16)	< **0.001** (42.20)	0.078 (3.39)
		Peyer's patches		
Star	1,22	0.301 (1.12)	0.139 (2.36)	0.718 (0.13)
Stard3	1,22	0.644 (0.22)	**0.002** (12.42)	0.619 (0.25)
Cyp11a1	1,20	0.592 (0.30)	0.564 (0.34)	**0.045** (4.57)
Hsd3b2	1,23	< **0.001** (23.89)	0.058 (3.99)	0.294 (1.15)
Nr5a2	1,22	< **0.001** (32.48)	**0.010** (7.90)	0.210 (1.67)
Hsd11b1	1,24	< **0.001** (23.42)	0.866 (0.029)	0.087 (3.18)
Hsd11b2	1,22	0.555 (0.36)	0.088 (3.18)	0.899 (0.02)

The data represent *p*-values with bolding indicating a statistically significant main effect or interaction effect; F values are given in parentheses; Df, degrees of freedom.
